# Application of FTIR Spectroscopy for the Elucidation of *Fusarium fujikuroi* Metabolites: New Insights in the Production of Organic Acids and Gibberellic Acid

**DOI:** 10.3390/jof12070527

**Published:** 2026-07-17

**Authors:** Aranza Hernández Rodríguez, Aarón Mendieta-Moctezuma, Raúl J. Delgado Macuil, Víctor Eric López y López

**Affiliations:** Centro de Investigación en Biotecnología Aplicada del Instituto Politécnico Nacional, Carretera Estatal Santa Inés Tecuexcomax-Tepetitla, Km 1.5, Tepetitla de Lardizábal, Tlaxcala 90700, Mexico; ahernandezr2201@alumno.ipn.mx (A.H.R.); amendietam@ipn.mx (A.M.-M.); rdelgadom@ipn.mx (R.J.D.M.)

**Keywords:** HPLC, FTIR, metabolites, batch culture, fungal fermentation

## Abstract

*Fusarium fujikuroi* is an industrial producer of gibberellic acid (GA_3_), a phytohormone of agricultural interest. Despite the high concentration of nutrients used for its production, GA_3_ yields remain low, highlighting the importance of identifying the major metabolites synthesized during GA_3_ synthesis. Therefore, the principal aim of this work was to evaluate organic acid production during *F. fujikuroi* batch cultures by determining GA_3_ production and organic acid profiles using Fourier transform infrared spectroscopy (FTIR) and liquid chromatography (HPLC) analysis. Significant differences in compound quantification were found; five organic acids, namely lactic, malic, citric, succinic and maleic, were detected by HPLC (in g/L: 101.09, 10.66, 2.80, 6.94 and 1.07, respectively). In addition, eight organic acids were determined by FTIR, namely lactic, butyric, pyruvic, fumaric, malic, succinic, maleic and oxalic (in g/L: 62.97, 19.19, 11.92, 7.54, 2.30, 4.36, 1.25 and 1.06 g/L, respectively). GA_3_ production was also quantified, reaching nearly 5.0 g/L as determined by HPLC and UV-Vis, and FTIR yielded 2.20 g/L. This report found that the low yields obtained in GA_3_ production are related to the side conversion of raw materials into organic acids as byproducts. In addition, the FTIR technique can be employed as an innovative strategy for the quantification of metabolites to provide relevant information on *F. fujikuroi* metabolic regulation. This enables the spread of its application as a biotechnological tool in high-value-added processes with potential for industrial-scale GA_3_ production.

## 1. Introduction

The gibberellins (GAs) are a large family of tetracyclic diterpenoid carboxylic acids discovered in the 1930s. GA compounds have a molecular structure containing between 19 and 20 carbon atoms comprising around 136 isoforms, but only certain gibberellins have functional bioactivities that give them the role of plant growth regulators; these are GA_1_, GA_3_, GA_4_, and GA_7_ [1,2]. Particularly, gibberellic acid (GA_3_) is a phytohormone of special interest in the agricultural sector, as its exogenous application induces positive effects in plants such as stimulation of cell division, seed germination, transition to flowering, sex expression, cell expansion, fruit development, and tolerance to abiotic stress [3,4].

Moreover, GA_3_ (C_19_H_22_O_6_) is a tetracyclic dihydroxy-γ-lactone acid that differs from other gibberellins in that it consists of a C1–C2 double bond and a C10 γ-lactone ring, two essential elements for its biological activity. This phytohormone is mainly synthesized by the secondary metabolism of *Fusarium fujikuroi*, from which industrial production is established. The synthesis of GA_3_ occurs via the terpene pathway and consists of three essential steps: (1) geranylgeranyl diphosphate (GGPP) conversion to *ent*-kaurene, (2) *ent*-kaurene conversion to GA_12_, and (3) GA_12_ conversion to different GAs [5,6]. Although the metabolic pathway of GA_3_ has been studied for several decades, there is still an information gap regarding the full set of metabolites that this fungal strain is able to produce. Multiple authors have described the synthesis of mycotoxins and pigments, such as beauvericin, carotenoids (β-carotene, lycopene, and neurosporaxanthin), bikaverin, fusaric acid, fusarubins, fusarin, and fusaric acid [7]. However, the reported yields are not proportional to the substrate ratios that are usually used to establish the fermentation process. For instance some of the best GA_3_ yields obtained through submerged fermentation represent only 3.62%, 3.40%, and 1.20% of the substrate utilization with respect to the total solids provided in the culture media (107.50 g/L [8], 64.10 g/L [9], and 173.81 g/L [2], respectively), while the metabolic direction of most of the carbon source is unknown.

Besides GA_3,_ it is necessary to identify the wide diversity of organic acids and other compounds synthesized during *F. fujikuroi* fermentation. This information would be helpful for obtaining a more comprehensive knowledge of the fungi’s metabolism, with the aim of developing efficient GA_3_ production processes. On the other hand, Fourier transform infrared spectroscopy (FTIR) has emerged as an innovative technique for characterizing molecular structures, which is based on the vibration and rotation of atoms. When a sample is irradiated with infrared light, the molecules with dipole moments absorb energy at specific frequencies, causing characteristic vibrational transitions and resulting in an absorbance spectrum that acts as a fingerprint [10]. In this way, FTIR has become a promising high-resolution technique that offers competitive advantages such as high reproducibility, a non-destructive nature and analytical speed, representing a suitable alternative to other conventional methodologies for monitoring fermentation processes; for example, companies like FOSS^®^ (MilkoScan^TM^, ProcesScan^TM^, ProFoss^TM^ DSoybean meal, ProFoss^TM^, NIRS^TM^ DS3 for oil processing, etc.) and Bruker^®^ (Tango II for food and beverage industry, feed manufacturing, pharma and biotechnology, chemical and petrochemical industry and polymer industry) develop equipment based on an infrared background for several industries to reduce time and materials and for use in line process. As far as the authors know, there are no reports about a specific determination of organic acid and GA_3_ production from *F. fujikuroi* fermentations at the bioreactor scale. Therefore, the major purpose of this research was to determine the organic acid profiles and GA_3_ yield produced by *F. fujikuroi* during batch cultures, using FTIR as the main analytical tool. This study contributes to the dynamic understanding of the metabolism of organic acids synthesis and GA_3_ production by *F. fujikuroi*, establishing the basis for the optimization and control of bioprocesses through monitoring with advanced spectroscopic tools.

## 2. Materials and Methods

### 2.1. Microorganism

*Fusarium fujikuroi* CDBB H-984 (also known as IMI 58289) was obtained from the Colección Nacional de Cepas Microbianas y Cultivos Celulares of the Centro de Investigación y de Estudios Avanzados del Instituto Politécnico Nacional (México City, México). This strain was stored in commercial potato dextrose agar (PDA) at 4 °C, and it was subcultured every 30 days for experimental purposes.

### 2.2. Culture Media

The composition of the culture media was modified from Escamilla et al. [8] to test a formulation medium with a C:N ratio of 50. Considering the carbon (C) content in rice flour (0.34 gC/g) and glucose (0.40 gC/g), as well as the nitrogen (N) content in ammonium chloride (0.262 gN/g), rice flour (0.0118 gN/g), and yeast extract (0.084 gN/g), the concentration of these components was modified to provide a medium with a C:N ratio of 50. Therefore, concentrations of carbon and nitrogen sources were adjusted to obtain this ratio as follows: glucose 100 g/L, NH_4_Cl 2.3 g/L, rice flour 5.5 g/L, KH_2_PO_4_ 3.0 g/L, MgSO_4_ 1.5 g/L, and yeast extract 2.0 g/L, given a total solid concentration of 114.30 g/L of the medium.

### 2.3. Batch Culture in Stirred Tank Bioreactor

Submerged fermentations were performed in a Sartorius Biostat^®^ Aplus (Göttingen, Germany) bioreactor with an operating volume of 4 L, inoculated in a ratio of 5% *v*/*v*. The operating conditions were as follows: temperature of 30 °C, pH of 5.0 (adjusted with NaOH 5M or H_3_PO_4_ 0.67M, as required), 300 rpm stirrer speed, and 1 vvm aeration rate (volume of air per volume of medium per minute). Samples were taken every 6 h over the fermentation period of 72 h.

### 2.4. Analytical Methods

#### 2.4.1. Biomass Quantification

The produced biomass was calculated by determination of the dry weight, where samples of 2 mL were filtered using a vacuum pump and filter paper (Whatman No. 1). The filters were dried to constant weight in an oven at 80 °C for 72 h. After incubation, the filter papers with and without samples were weighed and the difference resulted in the calculation of biomass production.

#### 2.4.2. Quantification of Glucose by the YSI Biochemical Analyzer

Samples of 2 mL were centrifuged at 12,000 rpm for 10 min to separate supernatants, which were collected to determine the glucose concentration profile (g/L) using the biochemical analyzer YSI 2700 SELECT (Yellow Springs, OH, USA).

#### 2.4.3. Quantification of GA_3_ by UV-Vis Spectrophotometry

The quantification of GA_3_ was based on the methodology described by Berríos et al. [11]. From this, 1 mL of supernatant samples was mixed with 1 mL of absolute ethanol in a 10 mL volumetric flask, then around 8 mL of HCl 3.75 M was added, and the mixture was vortexed for 10 s. The absorbance of the mixture was measured 20 s later at 254 nm using a 10 mm quartz cuvette to determine the concentration of GA_3_. The concentration was determined based on a standard calibration curve of GA_3_ ≥ 80% (Sigma-Aldrich^®^ Hamburg, Germany) within a sensitivity range of 0.1–1.0 g/L measuring absorbance at 254 nm (Thermo Scientific Multiskan GO, Waltham, MA, USA). The obtained data were adjusted using linear regression analysis (y = 0.0146 + 0.6450x, R^2^ = 0.99916) with the OriginPro 2021 software.

#### 2.4.4. Determination of GA_3_ by HPLC

The quantification of GA_3_ by high-performance liquid chromatography (HPLC) was based on the methodology described by Peng et al. [12], Sharma et al. [13] and Rangaswamy [14]. The HPLC system consisted of a Hewlett Packard Agilent 1100 HPLC System (Santa Clara, CA, USA) coupled to a diode array detector (DAD), equipped with a ZORBAX Eclipse XDB-C18 column (Agilent 961967-902, 4.6 × 150 mm, 5 µm, 400 bar). The mobile phase was a mixture of methanol/water at a ratio of 85:15. The injection volume was 0.5 µL, setting an isocratic injection flow of 0.80 mL/min and 30 °C temperature. Each sample had residence times of 5 min and detection at 210 nm (with an average retention time of 0.996 min for GA_3_). The samples were evaluated in a 1:1 dilution ratio. The phytohormone was quantified using a calibration curve of standard GA_3_ (Sigma-Aldrich^®^ ≥ 80%), prepared from solutions with concentrations ranging from 0.01 to 3.00 g/L. The results were analyzed by linear regression (y = 58614 + 7209.1x, R^2^ = 0.9931), using the OriginPro 2021 software.

#### 2.4.5. Determination of Organic Acids by HPLC

The quantification of organic acids by HPLC was based on the methodology described by Martínez et al. [15] and Scherer et al. [16]. The HPLC system was as described in the previous section. The analyzed samples were obtained from 2 mL of cell-free supernatants from fermentation samples at different times. As a general method, a mobile phase of 25 mM monopotassium phosphate (KH_2_PO_4_)/methanol (CH_4_O) was used at a volume ratio of 99:1 adjusted to pH = 2.6. The injection volume was 1.0 µL, setting an isocratic injection flow of 1.0 mL/min and 40 °C temperature. Each fermentation sample had residence times of 7 min and detection at 210 nm. The samples were evaluated in a 1:1 dilution ratio. The quantitative determination of lactic, malic, butyric, pyruvic, fumaric, succinic, oxalic, maleic, citric, acetic and isobutyric acid was performed using standard calibration curves for each organic acid (Sigma-Aldrich^®^ ≥ 99%) at concentrations ranging from 0.01 to 2.0 g/L. The obtained data were adjusted using linear regression analysis with the OriginPro 2021 software. The molecular structures of each organic acid are presented in Supplementary Material (Figure S1).

#### 2.4.6. Determination of GA_3_ and Organic Acids by FTIR Spectroscopy

As an alternative method for identifying and quantifying organic acids and GA_3_, spectroscopic measurements were performed in a Bruker VERTEX 70 FTIR infrared spectrometer (Bruker Optics Corporation, Billerica, MA, USA) in the Attenuated Total Reflectance (ATR) mode. For measurements, 1 µL of cell-free supernatant from each sample was placed on the ATR crystal, with a reading interval between 4000 and 400 cm^−1^, single scan time of 120 s and background scan time of 60 s. The obtained spectra were analyzed using the OriginPro 2021 software, identifying the characteristic peaks of each compound. The calibration curves were prepared for lactic, malic, butyric, pyruvic, fumaric, succinic, oxalic, maleic, citric, acetic, and isobutyric acids and GA_3_ at concentrations between 0.01 and 10 g/L, obtaining a linear regression analysis for each characteristic peak.

## 3. Results and Discussion

### 3.1. Growth Kinetics of F. fujikuroi During Batch Culture with a C:N 50 Ratio

**Figure 1:**
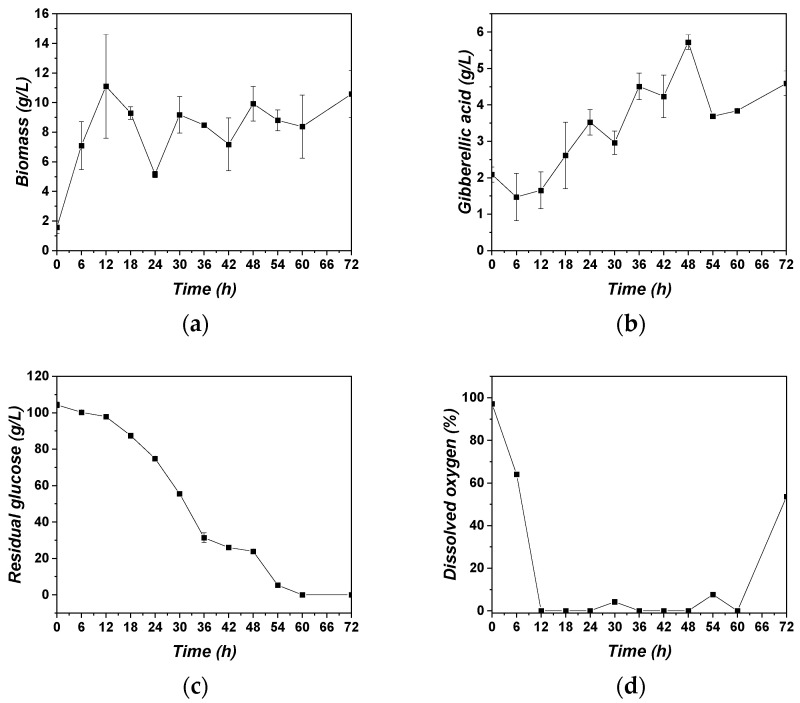

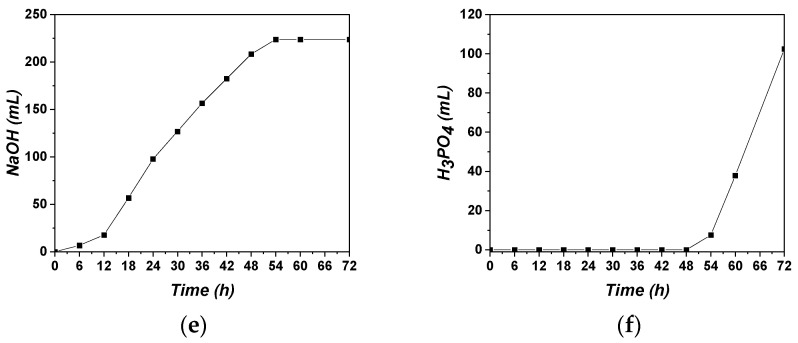
Growth kinetic profiles obtained from batch cultures at C:N 50 ratio. (**a**) Biomass production, (**b**) synthesized gibberellic acid, (**c**) residual glucose, (**d**) dissolved oxygen percentage, (**e**) base consumption (NaOH), (**f**) acid consumption (H_3_PO_4_).

Figure 1 presents the kinetic profiles of *F. fujikuroi* obtained during 72 h fermentations at bioreactor level. It was noticed that media at C:N 50 favored the growth of *F. fujikuroi*, but it did not present a classical growth curve. Instead, after reaching a maximal growth (11.10 g/L of biomass) at 12 h, the fungi enter in a sinusoidal behavior rather than the typical sigmoidal growth (Figure 1a).

Figure 1b shows the GA_3_ production profile determined by UV-vis spectrophotometry, where the maximal production was 5.72 g/L after 48 h fermentation. This behavior suggests that nitrogen-limited culture conditions, which means high C:N ratios, should be established to achieve maximum GA_3_ production, since its biosynthesis corresponds to a secondary metabolite that requires an unbalanced growth state [17]. Several authors describe that GA_3_ synthesis begins only when nitrogen exhaustion occurs, because nitrogen sources such as ammonium (NH_4_^+^) interfere with gibberellin production by suppressing the synthesis of gibberellin-involved enzymes, through a negative effect of ions [5,18,19]. In fact, it has been found that AREA (NIT2), a general transcription factor of GATA type zinc finger proteins, is involved in the de-repression of genes related to nitrogen metabolism for gibberellins biosynthesis in *F. fujikuroi* [20,21]. For example, the substitution of the *F. fujikuroi* homologues *areA*, *nit-2* and *areA-Gf* led to a significant reduction in gibberellin production yields [22]. Some studies show that deletion of *areA-Gf*—the major nitrogen regulator from *F. fujikuroi*—drastically reduces GA_3_ production. For example, the wild-type strain IMI 58289 produced 382.42 mg/L of GA_3_, while the *areA-Gf^-^* mutant strains showed a concentration of 100.90 mg/L, indicating a reduction of nearly 74% in gibberellin yield [23]. Accordingly, the expression of biosynthetic genes involved in gibberellin synthesis (*ggs2*, *cps/ks*, *des*, *P450-1*, *P450-2*, *P450-3* and *P450-4*) is reduced in mutants lacking *areA-Gf* [20,21]. This demonstrates that AREA not only participates in the regulation of genes involved in the assimilation of nitrogen sources but also plays a key role in the control of biosynthetic pathways for producing nitrogen-free secondary metabolites such as GA_3_ [20]. The GA_3_ production profile obtained from our batch cultures shows that the production of the phytohormone is minimal within the first stage of fermentation, consisting of lag and growth phases, since metabolism is primarily directed towards cell proliferation.

Residual glucose (Figure 1c) was totally consumed after 60 h, whereas dissolved oxygen (DO) (Figure 1d) decreased to zero at the maximal growth of *F. fujikuroi*, and it was maintained this way practically during the entire culture. For residual glucose determination, the standard deviation was very low, whereas for dissolved oxygen there was a unique value from the reactor sensor (no standard deviation).

In addition, the NaOH consumption rate (Figure 1e) showed an increasing behavior over time (from 6 until 72 h), demonstrating the progressive acidification from the batch culture, which could be associated with the production of organic acid compounds. Conversely, the H_3_PO_4_ consumption rate was lower, starting at 54 h (Figure 1f), as in the case of dissolved oxygen, for NaOH and H_3_PO_4_ determination was done with the reactor sensor (no standard deviation). This behavior suggests the major synthesis of extracellular metabolites by *F. fujikuroi*, capable of donating protons (H^+^) to the medium, causing an evident decrease in pH. This fact is not usually taken into account for the GA_3_ production from *F. fujikuroi*, despite the diverse substrates used in the media composition. Since these components are not converted into either biomass or gibberellic acid, we hypothesized the synthesis of a variety of acid byproducts such as organic acids.

These results not only demonstrate a significant improvement in the volumetric productivity and operational efficiency of this fungi system but also the reduction in fermentation times can also lead to reduced processing costs associated with energy and input consumption for further large-scale production.

### 3.2. Determination of GA_3_ and Organic Acids Production Profiles by HPLC

**Figure 2:**
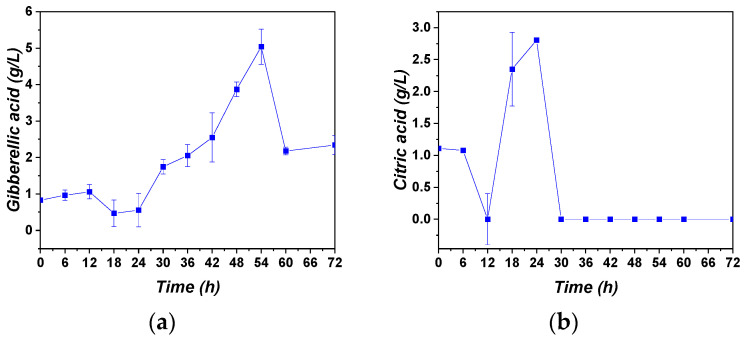

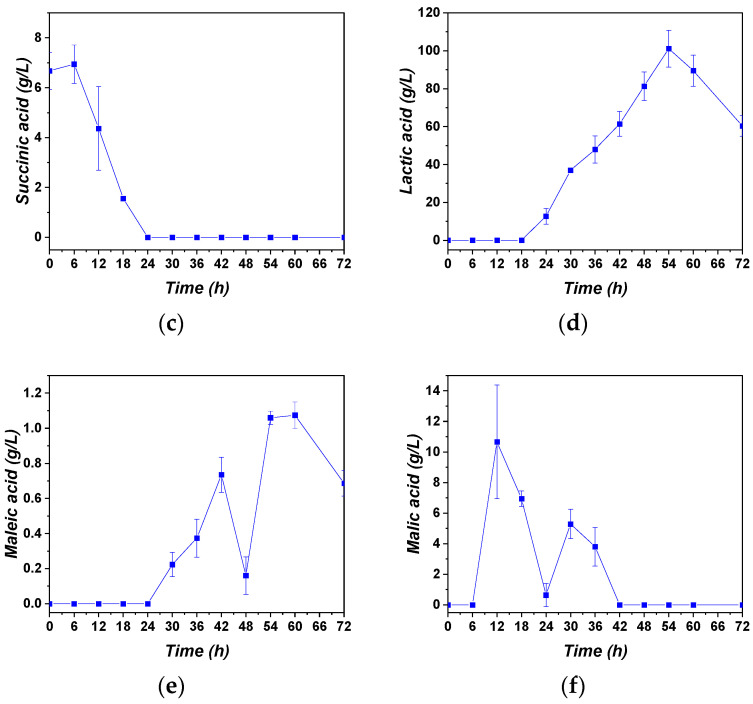
Organic acids and GA_3_ quantification by HPLC from samples obtained from batch cultures with C:N 50 ratio in bioreactor. (**a**) GA_3_, (**b**) citric acid, (**c**) succinic acid, (**d**) lactic acid, (**e**) maleic acid and (**f**) malic acid.

As shown in Figure 1b, the maximal GA_3_ production was 5.72 g/L after 48 h of fermentation, detected by UV-vis spectroscopy. Contrastingly, the concentration obtained by HPLC was 5.03 g/L at 54 h, highlighting an increase profile between 18 and 54 h followed by an abrupt decrease after (Figure 2a). This could be derived from instability and/or partial degradation of this metabolite. Other works have reported the GA_3_ quantification by the UV-vis obtaining a maximum production of 0.66 g/L of GA3 after 168 h of submerged fermentation [11] and obtained a maximum concentration of 0.82 g/kg of GA_3_ after 96 h of solid fermentation [24].

Regarding GA_3_ determination by HPLC, Escamilla et al. [8] reported a maximum production of 3.90 g/L after 192 h culture by *F. fujikuroi* CDBB H-984 (IMI 58289), the same strain used in this work. Also, Zhang et al. [2] achieved 2.10 g/L of GA_3_ at 168 h of fermentation with a mutant strain of *F. fujikuroi* (IMI 58289). According to the media formulation from these reports, the total solid nutrients concentration was calculated at 107.50 g/L for Escamilla et al. [8] and 173.81 g/L for Zhang et al. [2], compared to the 114.30 g/L solid nutrients used in this study. It is remarkable that, even with an improvement of GA_3_ production, the major nutrient consumption is not converted to the main product of interest.

Therefore, to improve the GA_3_ bioprocess, it is important to determine which are the major subproducts synthetized by *F. fujikuroi*, based on the evidence that organic acids are produced during the batch culture and are the logical candidates. Thus, an HPLC analysis was carried out to evaluate the retention time (RT) and characteristic signals of each organic acid detected at 210 nm (Table 1). The correct separation and identification according to the proposed method are shown [25,26].

Figure 2 presents the organic acid and GA_3_ production profiles determined by HPLC. From the 11 organic acids evaluated, only 5 were detected by this method, which are malic, succinic, citric, lactic, and maleic acids.

As shown in Figure 2, the organic acids that reached a concentration above 10 g/L were lactic and malic acids, reaching 101.09 g/L at 54 h and 10.66 g/L at 12 h, respectively. In contrast, the maximal concentrations obtained from the other organic acids were as follows: citric 2.80 g/L at 24 h, succinic 6.94 g/L at 6 h and maleic 1.70 g/L at 60 h. These results suggest a possible reorientation of the metabolic flux depending on the fermentation time and the C:N ratio of the culture media. Since a C:N 50 ratio appears to favor the secondary metabolism, it underlines the importance of considering the nutritional balance in the metabolic regulation of *F. fujikuroi*. The C:N ratio of a culture media significantly modulates the flux of the carbon source between primary and secondary metabolism, influencing the amount of an interest metabolite produced over fermentation time. This behavior suggests the activation of fermentative metabolic pathways, such as lactic fermentation, which induces the reduction of pyruvate to lactate as an energy conservation mechanism; under low oxygen concentration conditions, just as observed in our batch cultures within the interval from 12 to 60 h, the dissolved oxygen reached minimal levels at zero percent (Figure 1d) that correlates with the profile of lactic acid production. This relation suggests that, under hypoxic conditions, pyruvate does not enter completely to the Krebs cycle and the NAD^+^ generated is not reoxidized, so energy production is modified; NAD^+^ is regenerated from NADH by the reduction of pyruvate to lactate by the action of the enzyme lactate dehydrogenase [26]. Our results suggest that, under nitrogen-limiting conditions, as occurs in the media of C:N 50 used in this work, it promotes a reorganization of the carbon metabolism that is not focused on growth or energy production, yet it is mainly directed to typical compounds of the central metabolism rather than the ones from secondary metabolism [27,28] such as gibberellic acid.

### 3.3. Quantitative Determination of Organic Acids and GA_3_ by FTIR Spectroscopy

**Figure 3:**
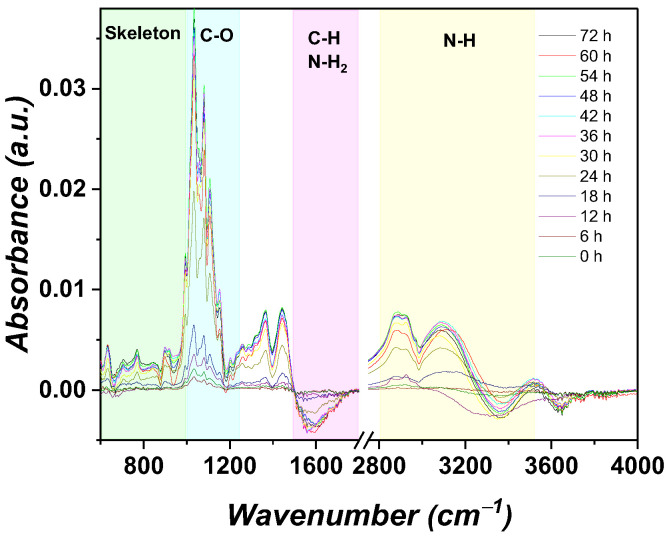
Infrared spectra of free-cell supernatants from samples of F. fujikuroi fermentation.

Figure 3 presents the infrared spectra of the samples selected from *F. fujikuroi* fermentations at C:N 50 ratio. This spectrum shows the change in absorbance intensity associated between samples compared to the initial of the fermentation process (0 h).

**Figure 4:**
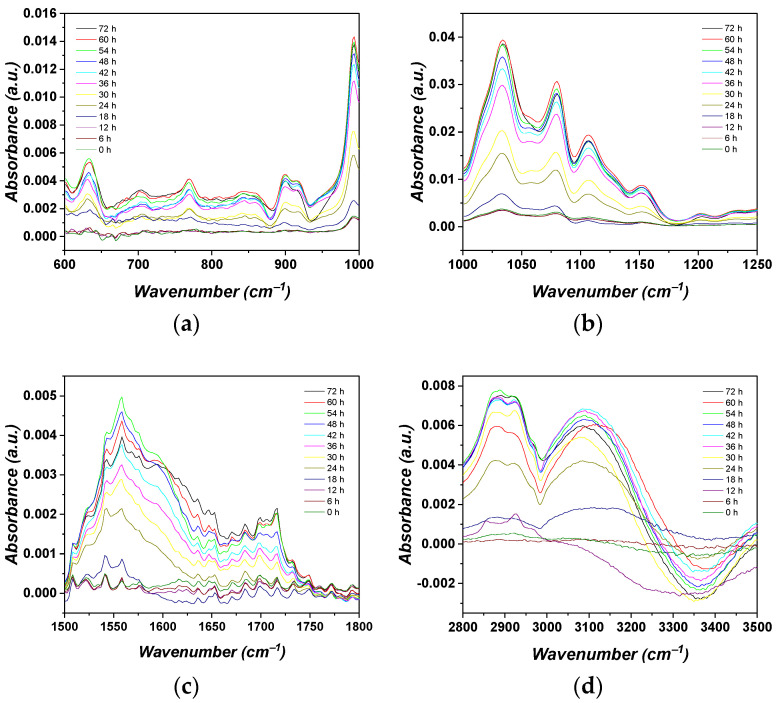
FTIR spectra of the different regions identified from peak signals obtained from samples if F. fujikuroi fermentation at C:N 50 ratio. (**a**) Molecular skeleton (600–1000 cm^−1^), (**b**) C–O (1000–1250 cm^−1^), (**c**) C–H y N–H_2_ (1500–1800 cm^−1^), and (**d**) C-H and N-H (2800–3500 cm^−1^).

The most evident changes were observed within the region corresponding to the molecule’s skeleton (600–1000 cm^−1^), C–O bonds (1000–1250 cm^−1^), C–H and N–H_2_ bonds (1500–1800 cm^−1^), C-H (2800–3000 cm^−1^), and N–H bonds (3000–3500 cm^−1^), which could be associated with the synthesis of organic acids and GA_3_ by *F. fujikuroi*. In the case of the region at 2800–3500 cm^−1^, it provides a reference for the protein contents in supernatants. These spectral regions are presented in Figure 4.

The molecule skeleton region at 600–1000 cm^−1^ (Figure 4a) shows an increasing intensity of the bands related to the 18 h samples and goes higher, reaching a maximum intensity at 60 h. Meanwhile, for the C–O region (1000–1250 cm^−1^), seven peaks characteristic of the region are defined, whose characteristic bands show a considerable increase in intensity after 24 h, reaching maximum absorbance at 60 h, as observed in Figure 4b. Then, Figure 4c shows a wide variety of peaks related to the C–H and N–H_2_ binding region (1500–1800 cm^−1^), with maximum intensity occurring at 54 h and an upward trend starting at 24 h. Lastly, Figure 4d shows a higher definition of three particular peaks in the N-H bond region (2800–3500 cm^−1^), where an upward trend in band intensity can be seen from 18 h, reaching maximum absorbance at 54 h. This region is mainly associated with nitrogen sources present in the culture medium (ammonium, peptides from yeast extract, proteins of rice flour, etc.), as well as proteins secreted by *F. fujikuroi*.

In general, the spectral behavior of the supernatants obtained from fermentation samples shows an increasing trend in the intensity of the bands of all chemical regions between 18 and 24 h, reaching maximum absorbances between 54 and 60 h. This behavior could result from the accumulation of carbonate, hydrogenate, oxygenate, and/or nitrogen compounds synthesized by the microorganism after the vigorous cell growth, transitioning from a morphological development stage to a production phase. The similarity in the times of the highest absorbance indicates the point of greatest biosynthetic activity, which is related to the production of primary and secondary metabolites by *F. fujikuroi*. Therefore, the spectral profile of fermentation samples supports the hypothesis of a time-dependent metabolic behavior, highlighting the points of metabolic redirection within the growth profile.

**Figure 5:**
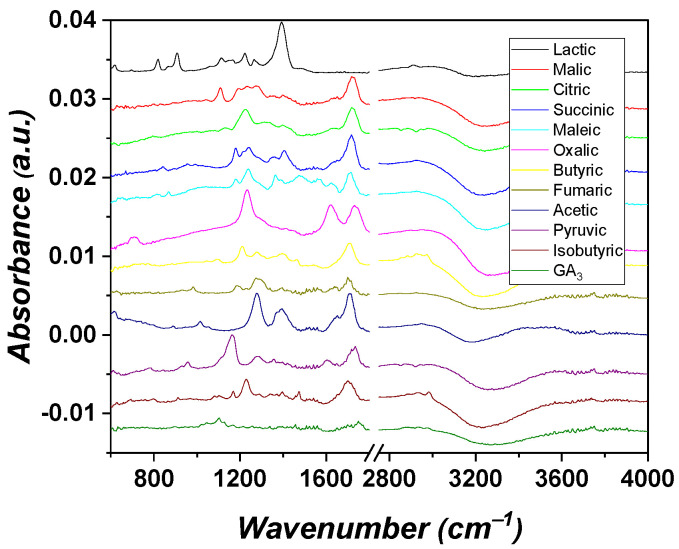
FTIR spectra of the organic acid standards at a concentration of 10 g/L.

The next step was to establish the spectral behavior of the standards of each organic acid and GA_3_ by FTIR spectroscopy (Figure 5). These spectra present the superposition of the infrared profiles obtained from the standards, which allowed the identification of particular signals from each compound, highlighting the suitability of FTIR spectroscopy to analyze all these standards.

**Figure 6:**
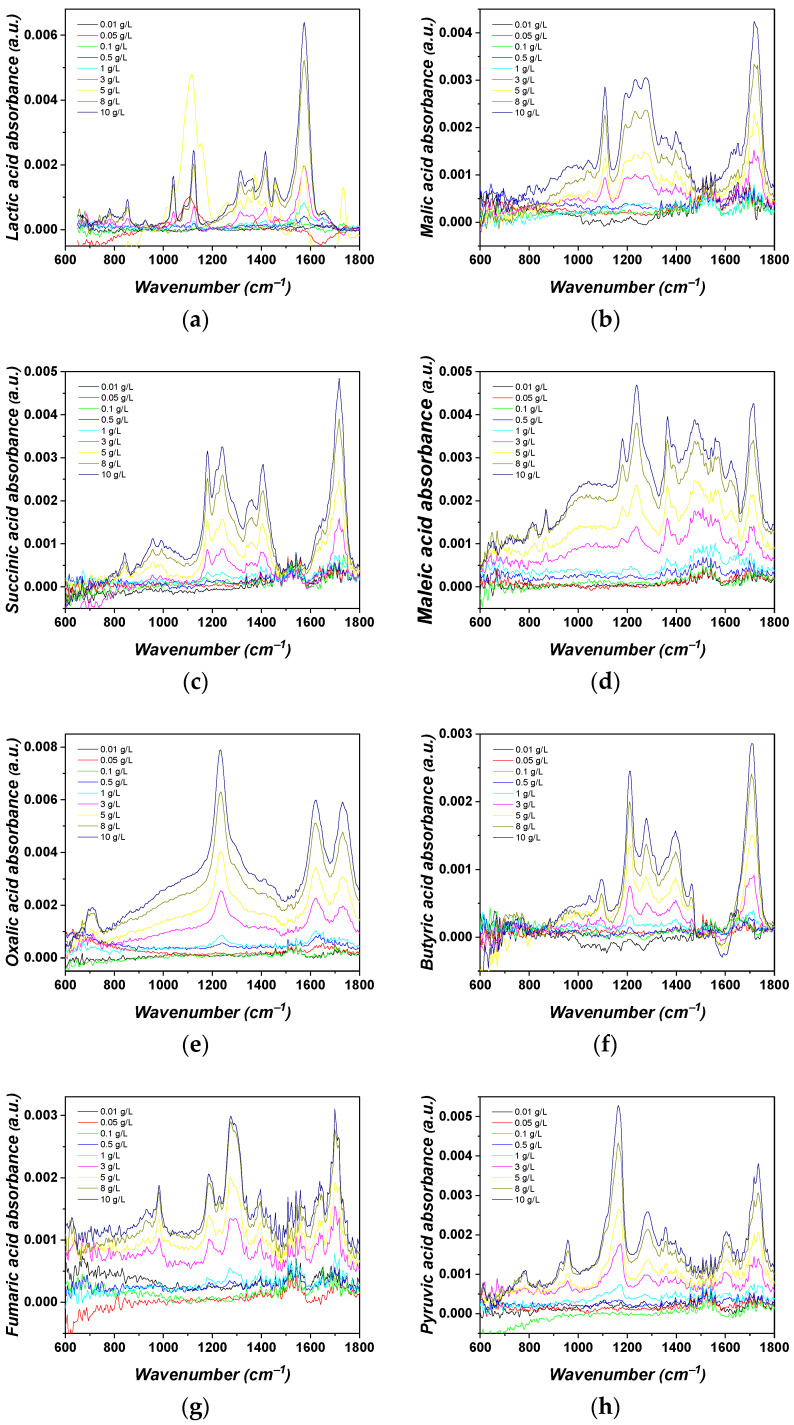

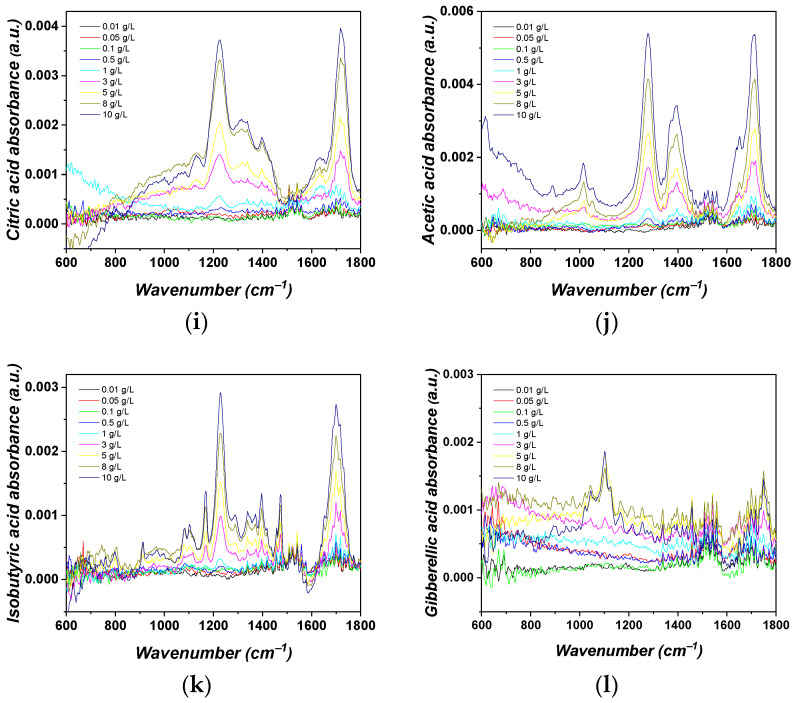
FTIR spectra behavior of organic acids and GA_3_ standards at different concentrations. (**a**) Lactic acid, (**b**) malic acid, (**c**) succinic acid, (**d**) maleic acid, (**e**) oxalic acid, (**f**) butyric acid, (**g**) fumaric acid, (**h**) pyruvic acid, (**i**) citric acid, (**j**) acetic acid, (**k**) isobutyric acid, and (**l**) GA_3_.

As observed, the spectral overlap of the standards shows differences between the peak pattern of the bands in the region between 600 and 1800 cm^−1^, with no significant changes in the region above 1800 cm^−1^. Since the region within 2800–3500 cm^−1^ did not correspond to organic acids and GA_3_, it was discarded from the analysis. Therefore, the spectral analysis will be focused only within the region between 600 and 1800 cm^−1^, as presented in the following sections. Thus, it is possible to establish the specific peak for each metabolite, laying the foundation for a strategy capable for the identification and quantification from fermentation samples. Since distinctive peaks were detected for each of the standards, the spectral behavior was evaluated at different concentrations as shown in Figure 6.

Figure 6a shows the spectral behavior of lactic acid, where changes in absorbance intensity were associated with the appearing peaks in the regions related to C–O (1000–1250 cm^−1^), C–C (1000–1350 cm^−1^), O–H (1250–1642 cm^−1^), C–H_3_ (1370–1470 cm^−1^) and C=O (1650–1850 cm^−1^) functional groups. This reflects a general tendency to increase the absorbance intensity as a function of the concentration. A similar behavior was observed for the spectral profiles of pyruvic (Figure 6h), acetic (Figure 6j), and isobutyric (Figure 6k) acids, which share the same functional groups as lactic acid.

In the case of malic acid, the spectral behavior (Figure 6b) showed variations in the intensity of the bands corresponding to the functional groups associated with C–O (1000–1250 cm^−1^), C–C (1000–1350 cm^−1^), O–H (1250–1642 cm^−1^), and C=O (1650–1850 cm^−1^) bonds. Similar patterns were observed for compounds that share functional groups analogous to malic acid, such as succinic, oxalic, butyric, and citric acids (presented in Figure 6c,e,f,i).

Moreover, Figure 6d shows the spectral profile of maleic acid, in which the observed peaks are associated with the regions of the C–O (1000–1250 cm^−1^), C–C (1000–1350 cm^−1^), O–H (1250–1642 cm^−1^), C=C (1620–1680 cm^−1^) and C=O (1650–1850 cm^−1^) functional groups belonging to the chemical structure of this compound, sharing similarity to the spectral regions of fumaric acid (Figure 6g).

Lastly, the infrared behavior of GA_3_ is shown in Figure 6l. The spectrum shows the characteristic peaks of C–O (1000–1250 cm^−1^), C–C (1000–1350 cm^−1^), O–H (1250–1642 cm^−1^), C–H (1330–1450 cm^−1^), C–H_3_ (1370–1470 cm^−1^), C=C (1620–1680 cm^−1^), and C=O (1650–1850 cm^−1^) regions, underlining that this compound showed most of the functional groups in its chemical structure, denoting the complexity of the GA_3_ molecule.

Regardless of the functional groups identified from samples, the spectral behavior of organic acids and GA_3_ showed a suitable tendency to increase the band absorption intensity as a function of the concentration, making it possible to differentiate high from low concentrations by FTIR. For a quantitative determination of these metabolites, we identified those peaks within the aforementioned regions that did not share spectral characteristics with the other organic acids, which allowed us to specifically distinguish each compound. Table 2 presents the unique peaks for each compound, which were used to generate a calibration curve with the most accurate correlation coefficient (R^2^).

These analyses validate the existence of specific spectral regions for each examined acid, ensuring the feasibility of metabolite discrimination in a bioreactor process, and also made it possible to generate linear correlation between absorbance intensity and compound concentration. In this way, each compound exhibited a distinct peak pattern, which ensured its selectivity. For example, of the seven peaks identified for maleic acid, none shared a spectral pattern with one another or with those of any other organic acid, thereby guaranteeing the specific identification of the compound.

To perform the quantitative determination of maleic acid using a calibration curve, the peak with the highest correlation coefficient was selected (peak at 1746 cm^−1^, R^2^ = 0.99). This ensured a strong proportional relation between the absorbance intensity of peaks and the compounds’ concentration, making the spectral band suitable for a quantification model with a high predictive potential. The same analysis was carried out for the rest of the compounds.

Table 3 presents the linear equation and the highest correlation coefficient (R^2^) derived from the calibration curves of the characteristic peaks for each organic acid, which are presented in Supplementary Material (Figure S2). This selectivity allowed the determination of signal peaks that exhibited a strong linear relationship between compound concentration and absorbance intensity, enabling high precision for the identification and quantification of organic acids in fermentation samples.

**Figure 7:**
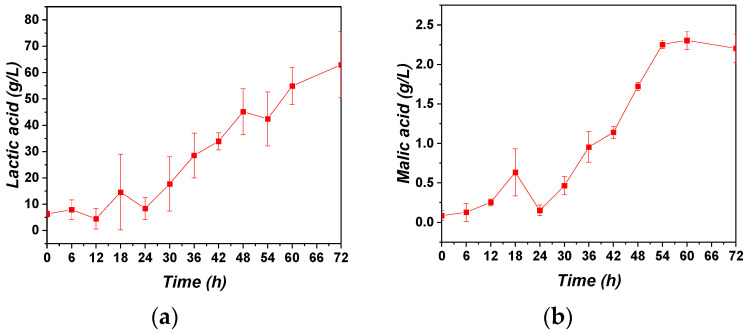

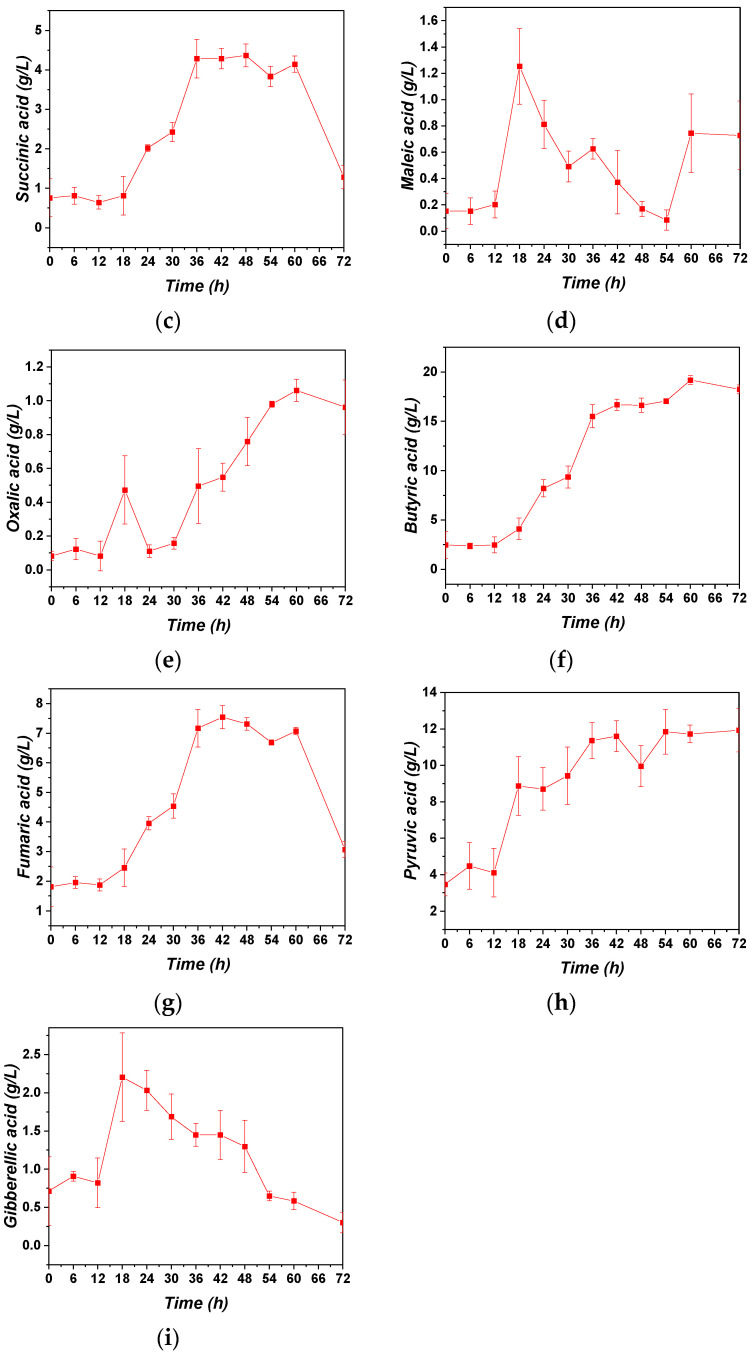
Production profiles of organic acids obtained from peak signal association by FTIR spectroscopy. (**a**) Latic acid, (**b**) malic acid, (**c**) succinic acid, (**d**) maleic acid, (**e**) oxalic acid, (**f**) butyric acid, (**g**) fumaric acid, (**h**) pyruvic acid and (**i**) GA_3_.

For lactic, acetic, citric and gibberellic acids, each one showed only one peak with a suitable linear trend at 1623, 1015, 1084 and 1749 cm^−1^, respectively. In contrast, succinic, fumaric, malic, isobutyric, pyruvic, oxalic, maleic and butyric acids exhibited two to seven peaks with a strong tendency to increase with concentration. Thus, the selected peaks were, respectively, 1180, 1184, 1724, 1418, 1490, 1731, 1746 and 1277 cm^−1^. Therefore, the production of each organic acid produced by *F. fujikuroi* was determined by FTIR spectroscopy, as shown in Figure 7. From the 12 standards evaluated, 9 were identified and quantified in the fermentation samples being lactic, malic, succinic, maleic, oxalic, butyric, fumaric, pyruvic acid and GA_3_.

The highest acid production was attributed to lactic acid (Figure 7a), as also determined by HPLC analysis. Since the first 12 h of culture are characterized by a stage of adaptation and cell growth, the concentrations of organic acids are minimal. During this period, metabolism is primarily directed toward the activation of primary metabolic pathways and biomass generation, limiting the synthesis of metabolites. This is supported by the observations of the growth profile (Figure 1a) and substrate consumption (Figure 1c), once the biomass concentration reached the highest level at 12 h, marking the key point of decline in substrate consumption. Therefore, subsequent carbon consumption is directed towards the production of acid compounds, as shown in all the kinetics. For instance, a pronounced increase in lactic acid production was observed from 18 h to 72 h, reaching a maximum concentration of 62.97 g/L. As mentioned before, this can be attributed to the greater nitrogen limitation that promotes the synthesis of the compound, possibly due to a high carbon availability that redirects the metabolic flow towards lactic acid fermentation. Furthermore, the low oxygen concentration conditions (Figure 1d) present in the culture medium encourage the activation of alternative fermentative metabolic pathways as an energy conservation mechanism. These results are important because they demonstrate the same trend observed by HPLC quantification (Figure 2d). Although the maximum concentration differs by 38%, both techniques establish that lactic acid is the predominant metabolite among all the quantified acids. In fact, there are no reports regarding such high lactic acid production by *F. fujikuroi*. Mostly, fermentation processes focus on the synthesis of the lactate from lactic acid bacteria; for example, Liu et al. [29] report one of the highest yields, with a production of 115.0 g/L of lactic acid by *Enterococcus mundtii*. In contrast, other authors report significantly lower yields, as in the case of El-Sheshtawy et al. [30], who obtained 28.14 g/L of lactic acid from *Kosakonia cowanii* bacteria, while Anagnostopoulou et al. [31] had a maximum concentration of 21.75 g/L with *Lactobacillus plantarum*. Even with the use of *Lactobacillus rhamnosus*, as described by Song et al. [32], only 30.25 g/L was obtained, representing calculated productivities of 0.39, 0.45 and 0.31 g/L·h, respectively. Comparing with our FTIR results, we obtained a 48% higher lactic acid concentration with a productivity of 0.87 g/L·h, exceeding the values specified in the studies mentioned. This demonstrates the efficiency of the developed fermentation system and places it among the bioprocesses with the highest reported yields, thereby helping to overcome the limitations established for *F. fujikuroi* fermentations. Therefore, the production of GA_3_ can be an integrative process to produce other value-added products such as lactic acid or others.

Figure 7b and Figure 7c show the malic and succinic acid production, respectively. After 12 h, an increasing trend is observed, reaching maximum values of 2.30 g/L at 60 h for the malic acid. According to the literature, it was found that West [33] obtained a malic acid production of 16.9 g/L at 192 h from a culture with *Aspergillus niger*. Cheng et al. [34] achieved a concentration of 31.30 g/L at 70 h with *Aureobasidium pullulans*, while Dörsam et al. [35] reached a production of 30.8 g/L at 168 h in a fermentation with *Aspergillus oryzae.* In contrast, for succinic acid the highest production was 4.36 g/L at 48 h. After these times, it was observed a pronounced decrease, reaching minimum values. On the other hand, the production of succinic acid relies mainly on the use of yeasts and bacteria. Regarding similar yields, Cui et al. [36] reported a succinic acid concentration of 12.05 g/L using *Yarrowia lipolytica*, while Louasté & Eloutassi [37] obtained a yield of 13.98 g/L with the *Actinobacillus succinogenes* strain. This could suggest the reuse of malic and succinic acids as metabolic intermediates for synthesis of other compounds, based on the *F. fujikuroi* metabolic and physiological needs.

This is relevant because it suggests that, although the yields achieved in this study are lower compared to the literature, this fermentation process triggers a metabolic dynamic that allows for the synthesis and subsequent reuse of succinic acid as an intermediate in primary metabolic pathways. This reflects the system’s capacity for producing the compound and contributes to the understanding of the effect of C:N ratio from the culture medium on the metabolism of *F. fujikuroi* over time. Comparing with the HPLC results, the profiles determined by both techniques differ in their behavior. This difference can be attributed to the analytical characteristics of each technique, signal interferences, variations in the concentration estimation, overlap of structurally similar compounds, and/or the sensitivity of the methodology, besides the nature of raw materials present in the medium cultures, such as glucose, rice flour, etc.

The maleic acid production profile is shown in Figure 7d, where a sharp production (1.25 g/L) was found at 18 h. After this time production was declined, suggesting a transient formation of maleic acid and a possible role as a metabolic intermediate in other metabolic pathways, the formation and consumption of which depends on the nutritional conditions of the medium. For this compound, FTIR showed a higher maximum concentration, as the value differed by only 14% between both techniques.

As we mentioned previously, oxalic, butyric, fumaric, and pyruvic acids were only detected by FTIR spectroscopy; therefore, the results obtained for these organic acids are highly relevant due to the productivity advantages offered by the evaluated fermentation process. For example, a maximum production of 1.06 g/L (60 h) was obtained for oxalic acid (Figure 7e), compared to that described by André et al. [38], who report a minimum concentration of 2.90 g/L (140 h) by *Aspergillus niger*. The calculated productivity was 0.017 and 0.020 g/L·h, respectively, indicating that there is a 15% discrepancy between both processes and that a similar production is achieved in a considerably shorter fermentation time in this research. In contrast, a maximum production of 19.19 g/L (60 h) was obtained for butyric acid (Figure 7f), while Jiang et al. [39] reported a concentration of 55.2 g/L (at 90 h) by *Clostridium tyrobutyricum*, resulting in productivities of 0.31 and 0.61 g/L·h, respectively. Similar results were obtained for fumaric acid (Figure 7g), estimating a maximum production of 7.54 g/L (at 42 h) was obtained, Wei et al. [40] reported a production of 4.67 g/L (96 h) by *Scheffersomyces stipitis*, corresponding to a process productivity of 0.17 and 0.04 g/L·h, respectively. This indicates that, in the present study, a 57% increase in fumaric acid concentration was achieved, with a 56% reduction (at 54 h) in fermentation time, demonstrating considerable efficiency under the established production conditions. On the other hand, the maximum production obtained for pyruvic acid (Figure 7h) was 11.92 g/L (at 72 h), somewhat similar to that described by Wang et al. [41], obtaining a concentration of 24.65 g/L (at 96 h) using a modified strain of *Saccharomyces cerevisiae*. The calculated productivities were of 0.16 and 0.25 g/L·h, respectively, which confers a discrepancy of 36%.

Figure 7i shows the production profile of GA_3_, characterized by a rapid synthesis from the initial stages followed by a gradual decrease toward the end of the fermentation, reaching a maximum concentration of 2.20 g/L at 18 h. This profile suggests that nitrogen-limited conditions promote the activation of secondary metabolic pathways related to gibberellin synthesis, and therefore the C:N ratio significantly influences the direction of carbon flux. Ei et al. [42] show gibberellin production by endophytic organisms and one isolate produces 94 ppm in 12th day of culture; however, this work was not focused on the quantification of the GA_3_ production by FTIR, and this was used as a verification of the presence of microbial GA_3_. Monrroy & García [43] reported a GA_3_ production of 6.1 g/kg in 11 days of process, which corresponds to only 0.6% of substrate converted in GA_3_, and FTIR technique was used for characterization of corn cob, not for GA_3_ determination. Omojasola & Adejoro [44] reported GA_3_ production by *Fusarium moniliforme* and *Aspergillus niger* using submerged fermentation of banana peel, using FTIR spectroscopy only to obtain the spectra of GA_3_ standard and GA_3_ extracted. These works used FTIR in transmission mode with KBr (after sampling processing); nevertheless, in our work, FTIR was used in the reflectance mode to determine the presence and simultaneous quantification of the major metabolites (not only GA_3_). At the same time, without sampling process in complex samples of fungal fermentation, this constitutes an innovative methodological contribution that significantly expands the analytical scope of FTIR beyond the detection of a single metabolite.

The results obtained by FTIR are comparable to those determined by HPLC. To the best of our knowledge, there are no reports comparing a chromatographic technique with a spectroscopic technique for quantifying the major metabolites from complex samples of fungal fermentation, highlighting the potential of FTIR as a robust, rapid, and non-destructive tool for monitoring bioprocesses. The concentrations obtained by FTIR are higher than those reported by various authors using the same microorganism. For instance, Camara et al. [1] obtained a production of 0.27 g/L of GA_3_ after 168 h of fermentation, while Rios-Iribe et al. [45] achieved a maximum concentration of 0.36 g/L after 288 h. Compared to the highest quantification obtained from our batch culture (2.20 g/L at 18 h), this represents a production increase of around 8.14 and 6.11 times, with a reduction in processing times of 89% (150 h) and 94% (270 h), respectively. Even with the application of genetically modified strain of *F. fujikuroi* [12], a maximum production of 2.8 g/L was achieved after 216 h of culture, indicating a comparable production (21% difference) but saving a total of 198 h of fermentation time (92%). This demonstrates a competitive advantage of the evaluated fermentation process, particularly attributed to the selected C:N 50 ratio, enabling an increased system productivity in considerably shorter processing times. Therefore, the operational efficiency places it as an outstanding alternative to previously reported processes, highlighting its potential for scaling up to different industrial levels.

Table 4 summarizes the organic acids determined by both FTIR and HPLC methodologies, showing the maximum yields and the corresponding fermentation times. As depicted, FTIR provided specificity, analytical sensitivity, detection range, and precision for tracking organic acid compounds present in the fermentation samples, allowing detection of nine acids.

The discrepancy observed in the identification of organic acids from both strategies can be attributed, to a large extent, to a peak overlap and co-elution phenomena found during HPLC analysis. The difference between the two techniques lies primarily in the identification of butyric, pyruvic, fumaric, citric, and oxalic acids. Pyruvic and fumaric acids were detected by FTIR, whereas by HPLC they were not detected. Analysis of the average retention times (RT) in HPLC shows a close proximity between the aforementioned compounds, with retention times of 0.972, 1.037, and 1.083 min for pyruvic, lactic, and fumaric acid, respectively. This proximity suggests a possible peak overlap between pyruvic and fumaric acids and lactic acid, which could lead to an overestimation in the concentration of lactate. Similarly, FTIR identified butyric acid, whose presence was not confirmed by HPLC. Although its retention time (4.162 min) differs from the aforementioned compounds, the structural similarity between butyric acid and lactic acid, including the same functional groups (C-H_3_, C=O, C-C, C-O, and O-H), similar molecular weights (88.106 and 90.078 g/mol, respectively), and high polarity, could increase the likelihood of analogous chromatographic behavior. Thus, similar affinities for the stationary phase could result in the elution of peaks at virtually the same time. Given these interactions, it is compressible that HPLC identified only lactic acid at a high concentration (101.09 g/L), unlike FTIR, which can discriminate from various organic acids, further quantifying butyric (19.19 g/L), pyruvic (11.92 g/L), fumaric (7.54 g/L), and lactic acid (62.97 g/L). Based on this, the sum of the concentrations obtained by FTIR (101.62 g/L) is comparable to the lactic acid concentration determined by HPLC (101.09 g/L), with a difference of less than 0.52%. These results suggest an overestimation of lactic acid by HPLC related to co-elution and peak overlap phenomena, arising from the structural similarity and chromatographic affinity of the analyzed compounds.

A similar pattern was observed between oxalic and citric acids. Oxalic acid was detected exclusively by FTIR, while citric acid was identified solely by HPLC. Although the retention times in HPLC are not similar, both compounds share the same functional groups (C-C, C-O, C=O, and O-H) and exhibit high polarity, which could influence their chromatographic behavior, specifically under conditions where the stationary phase is not sufficiently selective. This highlights the inherent limitations of HPLC technique in resolving structurally similar compounds. In contrast, FTIR allows for a broader and more specific differentiation of functional groups, revealing the simultaneous presence of several organic acids within a single sample.

Therefore, in this work we demonstrate that FTIR spectroscopy offers a significant advantage for the simultaneous monitoring of metabolites during fermentation processes using complex culture media, enabling the selective identification and quantification of various organic acids based on absorption bands from characteristic spectral regions. Nevertheless, both methodologies are complementary for the metabolic analysis of fermentation processes, and their analytical integration is key to ensure a reliable identification, enabling a broader monitoring and a comprehensive understanding of the metabolic profile, thereby avoiding biased interpretations resulting from the use of a single method.

## 4. Conclusions

*F. fujikuroi* possesses a significant metabolic capacity for the synthesis of diverse compounds of interest. However, the lack of knowledge regarding its metabolism considerably limits its application in profitable industrial processes. Our findings indicate that *F. fujikuroi* preferentially directs the conversion of carbon from raw materials toward primary metabolic pathways, favoring the synthesis of organic acids over the production of secondary metabolites such as GA_3_, even under conditions of a high C:N ratio. It evidenced the syntheses of malic, succinic, maleic, citric, oxalic, butyric, fumaric, gibberellic and lactic acids, obtaining a remarkable higher concentration of lactic acid during the fermentation process. Additionally, FTIR spectroscopy can be established as a reliable technique for the identification and quantification of organic acids in complex fermentation processes, such as GA_3_ production, showing that FTIR was a good alternative tool for metabolite determination. This research provides a deeper understanding of the metabolic dynamics of *F. fujikuroi*, offering a comprehensive view of its metabolic profile and the kinetic behavior of the compounds produced during fermentation.

This is important from an industrial perspective, as it achieves yields comparable to those of currently reported processes but with significantly shorter fermentation times, implying that substrate metabolism is focused on the synthesis of the compounds of interest. In this way, *F. fujikuroi* demonstrates its potential as a biofactory by establishing the basis for the optimization and scaling up of production processes for high-value-added compounds.

## Figures and Tables

**Table 1:** Retention times (RT) for organic acids and their characteristic peaks obtained by HPLC.

Organic Acid	RT Average (min)	TR Range (min)	
			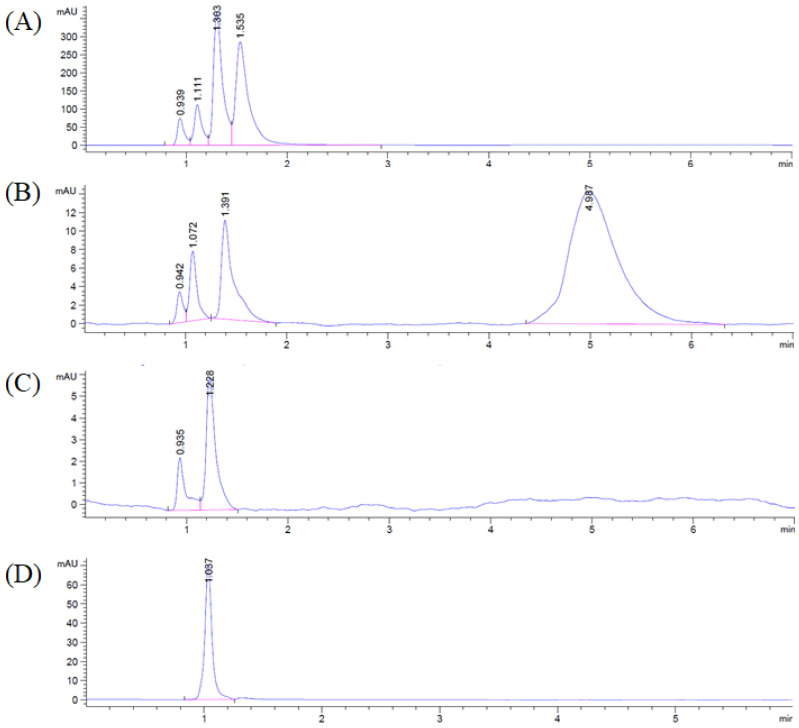
Oxalic	0.860	0.827–0.910	
Malic	0.992	0.940–1.010	
Lactic	1.037	1.012–1.090	
Acetic	1.131	1.120–1.150	
Maleic	1.130	1.090–1.120	
Citric	1.202	1.150–1.275	
Succinic	1.283	1.280–1.371	
Fumaric	1.083	1.375–1.425	
Butyric	4.162	4.091–4.400	
Isobutyric	4.197	3.942–4.896	
Pyruvic	0.972	0.910–0.940	
Gibberellic	0.997	0.996–1.037	
		

(A) Mixture of oxalic (0.939 min), lactic (1.111 min), fumaric (1.303 min) and maleic acid (1.535 min); (B) mixture of malic (0.942 min), citric (1.072 min), succinic (1.391 min), butyric and isobutyric acid (4.987 min); (C) mixture of pyruvic (0.935 min) and acetic acid (1.228 min); (D) GA_3_ (1.037 min).

**Table 2:** Absorbance peaks in the infrared spectrum characteristic of each organic acid and GA_3_.

Standard	cm^−1^	R^2^	cm^−1^	R^2^	cm^−1^	R^2^	cm^−1^	R^2^	cm^−1^	R^2^	cm^−1^	R^2^	cm^−1^	R^2^	cm^−1^	R^2^	cm^−1^	R^2^	cm^−1^	R^2^	cm^−1^	R^2^	cm^−1^	R^2^	cm^−1^	R^2^	cm^−1^	R^2^	cm^−1^	R^2^	cm^−1^	R^2^	cm^−1^	R^2^
Lactic																											**1623**	0.90						
Malic											1009	0.98			1194	0.99													**1724**	0.99				
Citric													**1084**	0.96																				
Succinic									955	0.98	991	0.98			**1180**	0.99																		
Maleic							813	0.97			1004	0.98	1085	0.98	1180	0.99			1388	0.99											1731	0.99	**1746**	0.99
Oxalic	694	0.98	710	0.96	719	0.96																									**1731**	0.99		
Butyric																	**1277**	0.99	1386	0.99														
Fumaric											982	0.95			**1184**	0.96																		
Acetic											**1015**	0.98																						
Pyruvic									957	0.98													**1490**	0.98	1604	0.97	1616	0.97						
Isobutyric													1081	0.97							**1418**	0.97												
GA_3_																																	**1749**	0.97

Pink rectangle: characteristic peak (cm^−1^); gray rectangle: correlation coefficient (R^2^); in bold: peak selected for each compound (cm^−1^).

**Table 3:** Linear equations and correlation coefficient (R^2^) derived from the calibration curves of the specific peaks for each organic acid analyzed by FTIR.

Organic Acid	Characteristic Peak	Linear Equation	R^2^
Lactic	1623	4.04931 × 10^−5^ x + 1.00018	0.907
Malic	1724	3.94925 × 10^−4^ x + 1.00015	0.998
Succinic	1180	3.12026 × 10^−4^ x + 1.00001	0.996
Maleic	1746	1.96913 × 10^−4^ x+ 1.00027	0.997
Oxalic	1731	5.71409 × 10^−4^ x + 1.00022	0.999
Butyric	1277	1.77682 × 10^−4^ x + 0.99998	0.993
Fumaric	1184	2.06448 × 10^−4^ x + 1.00019	0.967
Pyruvic	1490	8.27323 × 10^−5^ x + 1.00039	0.983
Citric	1084	9.56946 × 10^−5^ x + 1.00015	0.968
Acetic	1015	1.74941 × 10^−4^ x + 1.00000	0.985
Isobutyric	1418	5.53987 × 10^−5^ x + 1.00028	0.979
Gibberellic	1749	1.54153 × 10^−4^ x + 1.00044	0.973

**Table 4:** Maximum productions of organic acids and GA_3_ determined by FTIR and HPLC in the fermentation samples from bioreactor cultures with a C:N 50 ratio.

Organic Acid	FTIR		HPLC	
	Concentration	Time	Concentration	Time
	g/L	h	g/L	h
Lactic	62.97	60	101.09	54
Butyric	19.19	72	-	-
Pyruvic	11.92	72	-	-
Malic	2.30	60	10.66	12
Fumaric	7.54	42	-	-
Succinic	4.36	48	6.94	6
Gibberellic	2.20	18	5.03	54
Citric	-	-	2.80	24
Maleic	1.25	18	1.07	60
Oxalic	1.06	60	-	-

## Data Availability

The original contributions presented in this study are included in the article. Further inquiries can be directed to the corresponding author.

## Supplementary Materials

The following supporting information can be downloaded at https://www.mdpi.com/article/10.3390/jof12070527/s1, Figure S1: Molecular structure of each organic acid and gibberellic acid; Figure S2: Calibration curve of specific selected peaks for each organic acid and gibberellic acid.
