# Supplementary material for: Application of FTIR Spectroscopy for the Elucidation of Fusarium fujikuroi Metabolites: New Insights in the Production of Organic Acids and Gibberellic Acid
Source: J Fungi (Basel). 2026 Jul 17;12(7):527. doi: 10.3390/jof12070527 (PMC13413410; doi:10.3390/jof12070527)
Supplement: Supplementary file 1 [file jof-12-00527-s001.zip › jof-4423364-supplementary.pdf]

Supplementary Material

Application of FTIR Spectroscopy for the Elucidation of *Fusarium fujikuroi* Metabolites: New Insights in the Production of Organic Acids and Gibberellic Acid

Aranza Hernández Rodríguez, Aarón Mendieta-Moctezuma, Raúl J. Delgado Macuil and Víctor Eric López y López *

Centro de Investigación en Biotecnología Aplicada del Instituto Politécnico Nacional, Carretera Estatal Santa Inés Tecuexcomax-Tepetitla, Km 1.5, Tepetitla de Lardizábal, Tlaxcala 90700, Mexico; ahernandezr2201@alumno.ipn.mx (A.H.R.); amendietam@ipn.mx (A.M.-M.); rdelgadom@ipn.mx (R.J.D.M.)
*Correspondence: vlopezyl@ipn.mx

Supplementary material

Molecular structure of organic acids and gibberellic acid

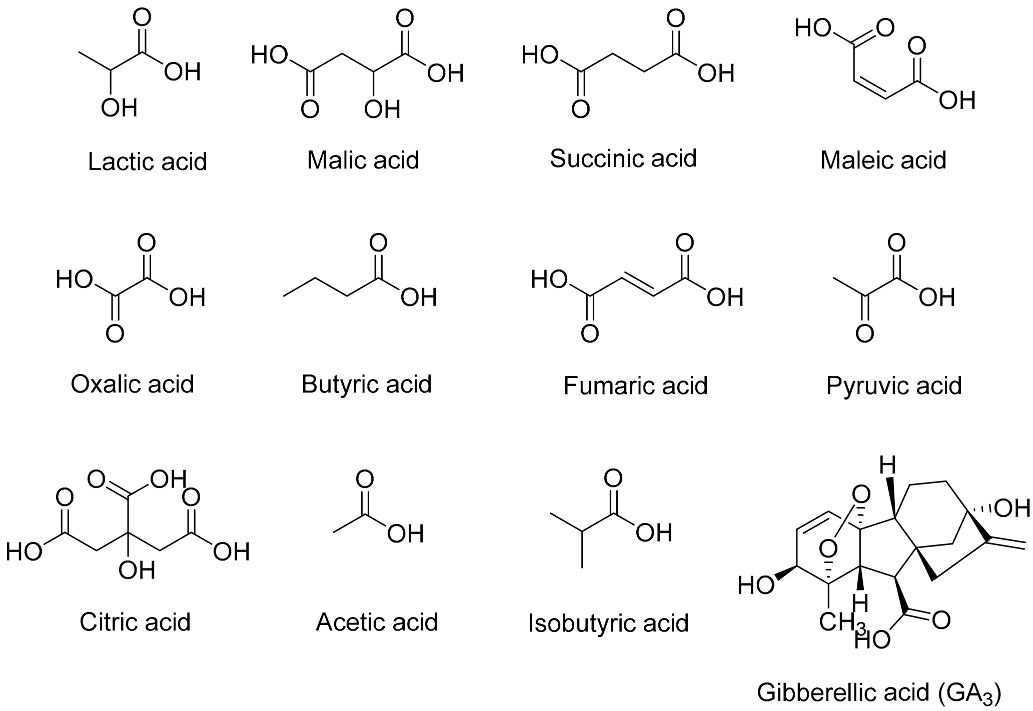

Figure S1. Molecular structure of each organic acid and gibberellic acid.

Calibration curves of organic acids and gibberellic acid

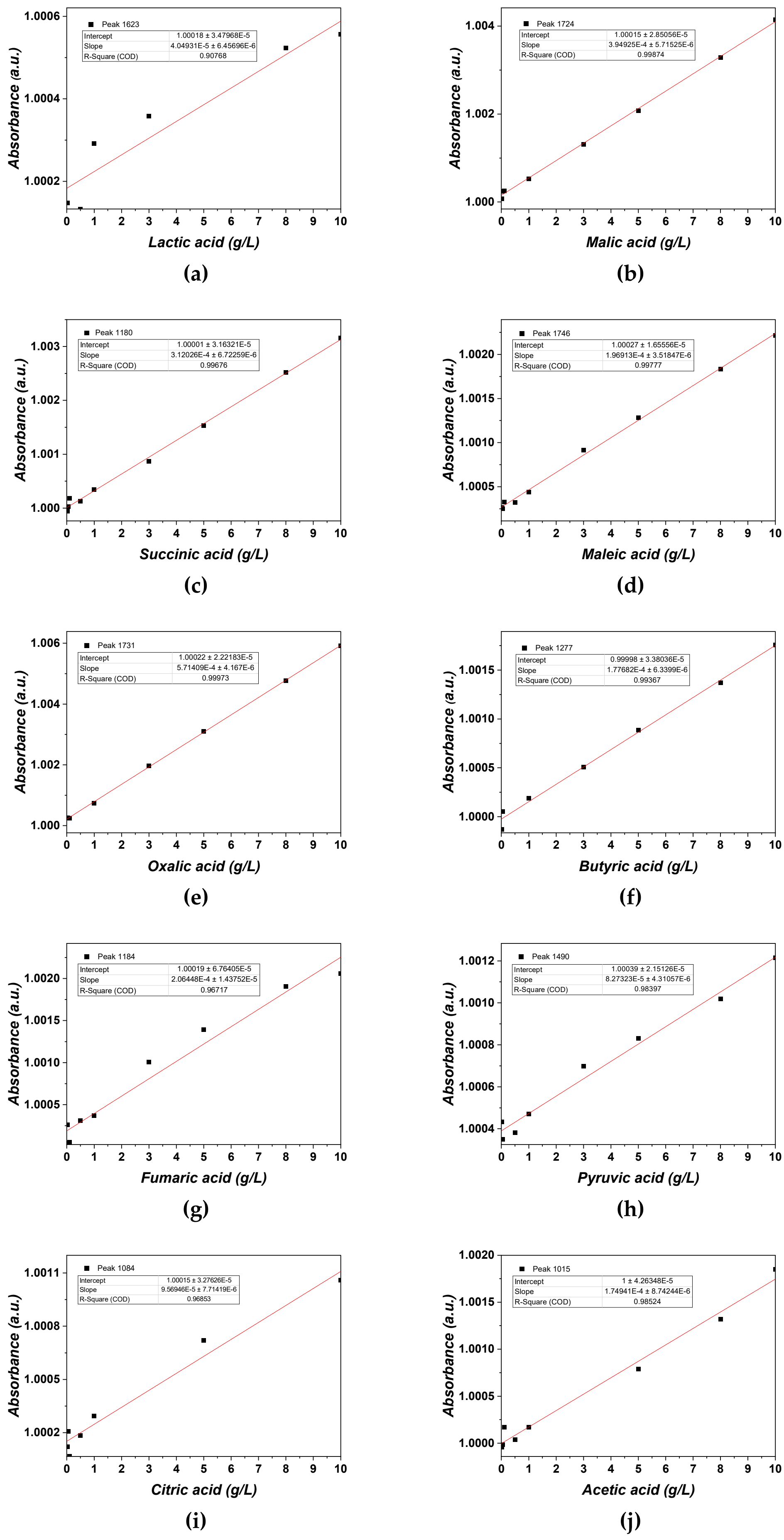

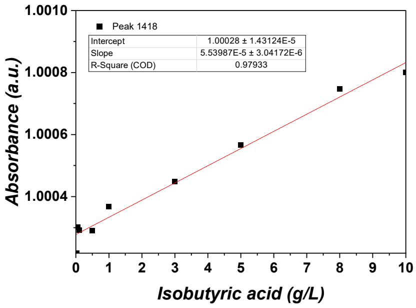

(k)

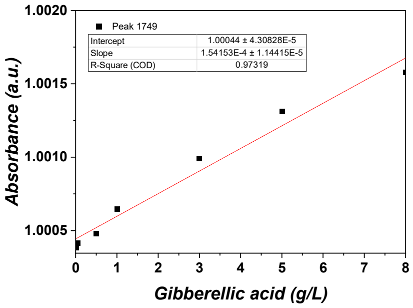

(l)

Figure S2. Calibration curve of specific selected peaks for each organic acid and gibberellic acid. (a) Lactic acid, (b) malic acid, (c) succinic acid, (d) maleic acid, (e) oxalic acid, (f) butyric acid, (g) fumaric acid, (h) pyruvic acid, (i) citric acid, (j) acetic acid, (k) isobutyric acid, and (l) GA_3.
